# Creative music therapy in preterm infants effects cerebrovascular oxygenation and perfusion

**DOI:** 10.1038/s41598-024-75282-8

**Published:** 2024-11-15

**Authors:** Felix Scholkmann, Friederike Haslbeck, Emily Oba, Tanja Restin, Daniel Ostojic, Stefan Kleiser, Bartha C. H. Verbiest, Hamoon Zohdi, Ursula Wolf, Dirk Bassler, Hans Ulrich Bucher, Martin Wolf, Tanja Karen

**Affiliations:** 1 Biomedical Optics Research Laboratory, Department of Neonatology, University Hospital Zurich, University of Zurich, Zurich, Switzerland; 2 Neurophotonics and Biosignal Processing Research Group, Biomedical Optics Research Laboratory, Department of Neonatology, University Hospital Zurich, University of Zurich, Zurich, Switzerland; 3 Newborn Research Zurich, Department of Neonatology, University Hospital Zurich, Zurich, Switzerland; 4 Institute of Complementary and Integrative Medicine, University of Bern, Bern, Switzerland; 5 Neuroscience Center Zurich, University of Zurich and ETH Zurich, Zurich, Switzerland; 6 Institute of Physiology, University of Zurich, Zurich, Switzerland; 7 OxyPrem AG, Zurich, Switzerland; 8 Lucerne Cantonal Hospital, Lucerne, Switzerland; 9 Department of Information Technology and Electrical Engineering, ETH Zurich, Zurich, Switzerland

**Keywords:** Optical neuroimaging, Functional near-infrared spectroscopy, fNIRS, Creative music therapy, Preterm infants, Brain development, Development of the nervous system, Neuro-vascular interactions, Social neuroscience

## Abstract

Creative music therapy (CMT) has been shown to promote the development of brain function and structure in preterm infants. We aimed to investigate the effect of CMT on cerebral oxygenation and perfusion to examine how the brain reacts to CMT. Absolute levels of cerebrovascular oxygen saturation (StO_2_) were measured in clinically stable preterm-born neonates (*n* = 20, gestational age: ≥30 weeks and < 37 weeks) using two near-infrared spectroscopy (NIRS)-based tissue oximeters over the right prefrontal cortex and left auditory cortex. We applied the systemic physiology augmented functional NIRS approach. Each CMT session lasted 55 min and involved 9 intervals, including two 10-minute intervals during which the music therapist hummed and held the neonate. We found that CMT-induced changes in cerebrovascular StO_2_, perfusion and systemic physiology (i) could be classified into two groups (group 1: increase in StO_2_ during the first singing interval, group 2: decrease in StO_2_), (ii) differed in female neonates compared to male neonates, and (iii) correlated with individual blood haematocrit levels. Our exploratory study (i) demonstrates the impact of CMT on the neonate’s physiology and (ii) highlights the need to analyze functional NIRS measurements in neonates separately according to their response pattern to avoid erroneous conclusions, e.g. when only the group average of the signal change is determined.

## Introduction

According to the most recent analysis (based on data from 2014) about the global incidence of preterm birth, an estimated 15 million premature babies (< 37 weeks gestation) are born worldwide each year (representing 10.6% of live births), with more than 80% occurring in Asia and sub-Saharan Africa^1^. Improvements in medical care in recent decades have resulted in more infants surviving, but short- and long-term neurodevelopmental impairments remain constant, placing an ongoing burden on families, society and medical institutions^2–4^.

Very premature infants spend their first time of life in the neonatal intensive care unit (NICU), where they are exposed to the noise of the NICU. On the one hand, this noise can make it difficult to hear the voice of the caregiver, and, on the other hand, it is a direct acoustic stressor. Our group has recently shown that the neonatal incubators themselves are a source of noise and at the same time do not adequately protect the newborns sufficiently from the noise of the NICU^5^. One source of noise, for example, is false alarms from patient monitors—a problem that is now being addressed by improvements in technology and algorithms^6,7^. Previous research has focused on how music and vocal interventions can enhance the auditory and multisensory experiences of infants and their parents during this sensitive and valuable phase of neurodevelopment^8^. Music-based interventions in neonatal care^9–11^ have been shown to positively influence infant vital signs, maternal anxiety, oral feeding volume and stress levels, with moderate-to-high heterogeneity between studies^12–14^.

One approach to live music therapy in neonatal care is Creative Music Therapy (CMT)—a personalized, non-invasive, non-pharmaceutical, interactive, resource-oriented and family-integrating therapy^15–17^. CMT emphasizes that being responsive to music is an intrinsic quality of being human, no matter how ill, disabled, or even premature an infant may be^18,19^. In CMT, the therapist assesses the meaning of the infant’s breathing patterns, facial expressions and gestures to construct a musical response with humming and singing in lullaby style, as infant-directed humming and singing is considered the most natural and attractive musical-social stimulus for infants^20–22^. CMT has been shown to improve neurodevelopment in preterm infants^23^. CMT may have a positive effect on functional connectivity of the brain as measured by resting-state functional magnetic resonance imaging (fMRI), with improved thalamocortical processing, functional networks and functional integration in the left inferior temporal, prefrontal and additional motor and brain areas.

Although the effect of music and singing interventions on neonatal cerebrovascular oxygenation has been studied in the past, a real-time assessment of the potential immediate effects of CMT on brain activity in preterm infants, in particular how auditory and tactile stimulation affect cerebrovascular oxygenation, perfusion and metabolism, has not yet been investigated. Among brain imaging technologies, functional near-infrared spectroscopy (fNIRS) appears to be the most suitable for this purpose, as it is a silent, non-invasive, portable technology for assessing evoked changes in cerebrovascular oxygenation and hemodynamics^24–26^, especially for clinical and research use in preterm infants^27–29^. In particular, fNIRS studies of auditory stimulation in preterm and newborn infants have demonstrated stimulus-evoked changes in cerebrovascular oxygenation and hemodynamics in frontal areas^30–34^ and temporal cortices^34–36^ (for a review see de Roever et al.^37^). In addition, it has been suggested that the left hemisphere dominates overall sound processing in neonates^38^—a finding supported by an fMRI study from our group showing a left lateralization of brain activity affected by CMT, particularly in left inferior temporal brain regions^23^.

In two recently published fNIRS studies investigating the effect of music on brain activity in preterm infants, increases in cerebrovascular oxygenation were observed during live music therapy (instrumental and vocal)^39^ and during maternal singing combined with skin-to-skin care^40^.

Improving stimulus-evoked changes in oxygenation and perfusion in the auditory and prefrontal cortex is critical after preterm birth, as preterm infants are at risk for neurodevelopmental impairment, and the plasticity of auditory regions and cortical development are highly dependent on the quality of early auditory experiences^41^. According to a recent systematic review and meta-analysis of randomized controlled trials (*n* = 13) on the effect of music interventions in preterm infants in the NICU^42^, the authors concluded that music interventions “significantly improve preterm infant’s heart rate, respiratory rate, and stress level, as well as increase oral feeding volume” which “may exert a positive impact on well-being and quality of life in preterm infants” in the NICU. However, a recent Cochrane systematic review conducted by members of our group (FH, TK, DB)^43^ concluded that music and vocal interventions were not associated with a substantial increase in cerebrovascular oxygenation during or after the intervention with high-certainty evidence.

Therefore, the aim of our new fNIRS study was to investigate in detail whether CMT (as routinely performed at the NICU of the Department of Neonatology, University Hospital Zurich) alters cerebrovascular oxygenation and perfusion in the left auditory and frontal cortex of preterm infants, and how this relates to baseline and evoked changes in systemic physiological parameters. Our exploratory study aimed to better understand how the brain responds to CMT in preterm infants.

## Subjects and methods

### Participants

Clinically stable (i.e. with stable vital signs as judged by the clinician in charge) preterm infants (*n* = 20, gestational age: ≥30 weeks and < 37 weeks) without any additional respiratory or oxygen support and with parental informed consent were included in our study. Infants aged < 30 weeks were not chosen, as this population tends to be less clinically stable and their head size would have been too small for optimal placement of our NIRS oximetry sensor. Infants with congenital malformations were excluded. None of the included infants had been exposed to CMT prior to enrolment. As this was an exploratory study, the sample size for the study design could not be calculated directly (as the effect size was unknown). The number of subjects was chosen to be in the range of similar fNIRS studies in neonates performed by our group^27,44–46^ and for practical reasons, i.e. to perform the difficult measurements in the NICU and the difficult recruitment of subjects within a realistic time frame.

The study was conducted in 2021 at the Department of Neonatology of the University Hospital Zurich, and approved by the ethical committee of Zurich (KEK 2010-0102/2) and Swissmedic (2010-MD-0019). All methods were performed in accordance with the relevant guidelines and regulations of the University Hospital Zurich and ethical committee of Zurich.

Due to insufficient data quality or technical measurement errors, 3 data sets (i.e. 15%) had to be excluded from further analysis. The final data set used for the analysis included measurements from 17 preterm-born neonates (gestational age (median) [interquartile range, IQR]: 33.7 weeks [3.5 weeks], postnatal age: 9.0 days [9.0 days]; 7 males, 10 females; birth weight: 1730 g [570 g]). No adverse events occurred during the study period.

### Study protocol

The measurements were carried out in a quiet family room near the NICU. Once the equipment was installed and the respective neonate was in a calm state (no crying, no strong movements, no restlessness), the experiment began. During the measurements, the room lighting was dimmed to avoid possible interference of the ambient light with the fNIRS measurements and to create a more comfortable atmosphere for the neonates. At least one parent was present during the experiment, observing the proceedings but not actively involved.

**Fig. 1:**
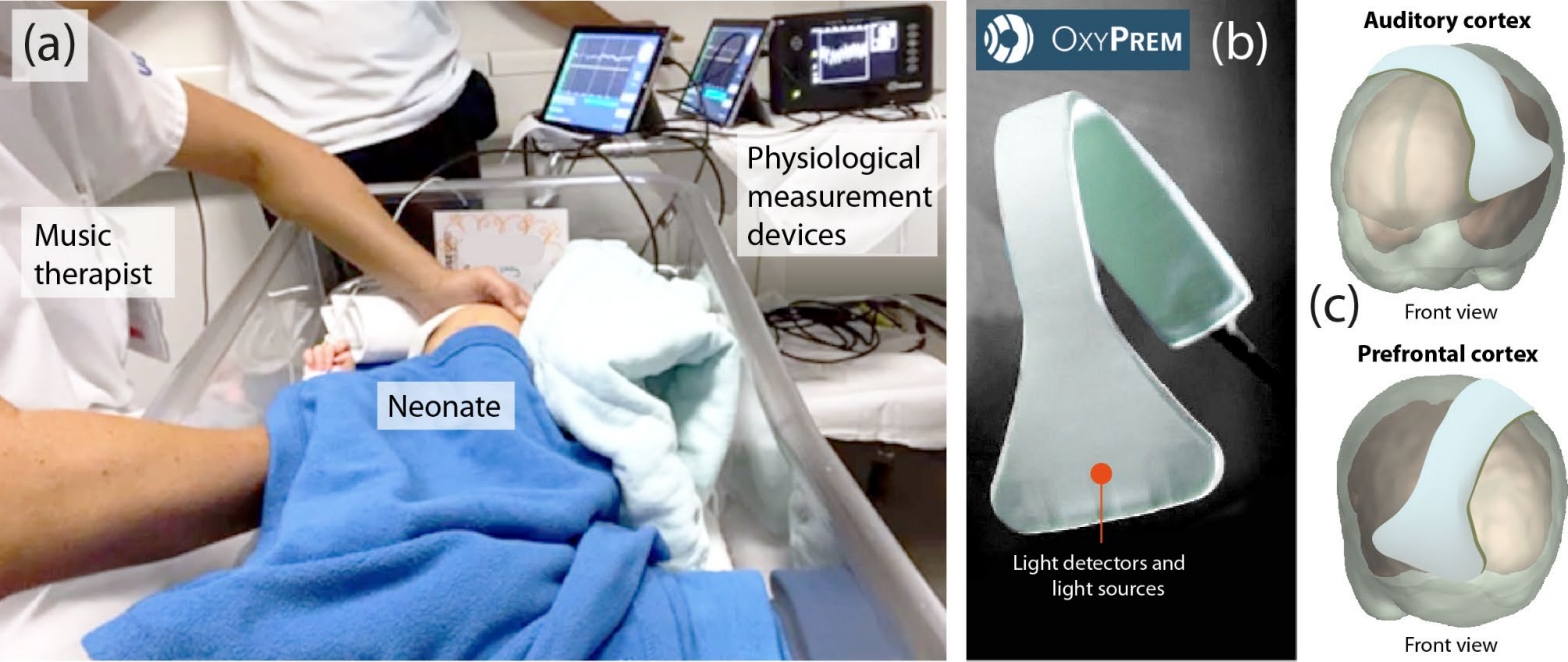
Measurement setup. (**a**) Picture of a measurement session where the music therapist touches the child with both hands while measuring StO_2_, SpO_2_ and PR. (**b**) One of the OxyPrem v1.4 NIRS-based oximeters used for the fNIRS measurements. (**c**) Visualization of the NIRS optode placement on the neonate’s head. (The neonatal head model is based on an fMRI scan of a 35-week-old infant as available in the AtlasViewer software^56^).

The experimental protocol was as follows: After a baseline period (5 min), the CMT therapist made contact with the infant for 5 min by holding the back of the infant’s head with her left hand and the base of the infant’s head with her right hand, which is defined as the first therapeutic touch in the CMT clinical protocol^47^ (see Fig. 1a). After assessing the infant`s gestures, facial expressions and breathing, the therapist began with the gentle humming of B3s and C4s, matching the infant’s breathing rhythm, gestures and facial expressions, and after about 30 s changed to humming the traditional German lullaby “Schlaf Kindchen schlaf” (Sleep, baby, sleep) in B major (see Appendix Video 1). She continued to touch the infant and hummed the lullaby calmly and slowly for 10 min, improvising flexibly to adapt the melody to the infant`s behavioural state. After 9 min, she gently let the humming subside, stopped humming after 10 min, and held the infant for another 5 min. This sequence was then repeated to investigate a possible habituation effect on the second exposure. In total, the whole sequence lasted 55 min and consisted of 9 intervals. The behavioural states of the newborn were closely observed at the bedside according to Prechtl’s criteria^48^.

### SPA-fNIRS measurement setup

Absolute values of cerebrovascular oxygenation (StO_2_) were measured using two near-infrared spectroscopy (NIRS)-based tissue oximeters (OxyPrem v1.4, OxyPrem AG, Zurich, Switzerland) (Fig. 1b). The NIRS optode of the first device was placed over the right prefrontal cortex (PFC) and the second optode over the left auditory cortex (AC) (Fig. 1c). To prevent slippage and to ensure good contact between the skin and the sensor surface, the optodes were fixed with an elastic band (DermaPlast CoFix, IVF Hartmann AG, Neuhausen).

Equipped with two light sources (each with eight light-emitting diodes [630–980 nm] and symmetrically arranged light detectors), the OxyPrem 1.4 allows an accurate and robust determination of StO_2_ while operating in continuous wave (CW) mode and using a self-calibrating multi-distance (SCMD) measurement technique—as confirmed in a previous study (using version 1.3 of the device), which showed excellent measurement precision (≤ 1.85%)^49^. The SCMD measurement technique reduces the likelihood of measurement artifacts caused by changes in light coupling between the skin and the light sources and the detectors, influences from extra-cerebrovascular oxygenation and perfusion, and movement of the tissue relative to the sensor^50^. The depth-resolved measurements allow for greater sensitivity to cerebral tissue compared to extracerebral tissue, which is necessary to assess the neurovascular coupling associated with brain activity^51^.

The SenSmart-X100 patient monitor (Nonin Medical, Inc., Plymouth, Minnesota) was used in parallel to measure peripheral arterial oxygenation (SpO_2_) and pulse rate (PR) by attaching the neonatal pulse oximetry sensor to the neonate’s right hand. StO_2_ time-series were recorded with a sampling frequency of 1 Hz, the SpO_2_ and PR time-series with 0.25 Hz.

From the recorded StO_2_ and SpO_2_ time-series, the time-dependent fractional tissue oxygen extraction (FTOE) was calculated (FTOE = (SpO_2_–StO_2_)/SpO_2_)^52^, an index for the balance between oxygen supply and oxygen consumption in tissue.

With the two NIRS-based oximeters and the patient monitor assessing the brain-related and systemic physiological state of the neonate in parallel, a measurement approach called “systemic physiology augmented functional near-infrared spectroscopy” (SPA-fNIRS) was realized, which allows a comprehensive physiological interpretation of the fNIRS measurements^53–55^.

### Signal processing and statistical analysis

The StO_2_ time-series were first downsampled to the same sampling frequency (0.25 Hz) as the peripheral physiological time-series (SpO_2_, PR) were recorded. A finite impulse response (FIR) antialiasing low-pass filter was used for this. To remove high-frequency noise and fluctuations of no interest, the SpO_2_ and PR time-series were smoothed using a Savitzky-Golay (SG) filter of 2nd degree and a 32 s window. Downsampling and low-pass filtering did not remove possible physiological information of interest, as the changes in cerebrovascular hemodynamics and oxygenation relevant to the current study occur in the frequency range < 0.25 Hz.

To remove the motion artefacts from the data, an approach similar to the methods previously developed by our group^57,58^ was used: Acceleration data in the spatial directions *x*, *y* and *z*, also measured by the NIRS instrument, were summed and the moving standard deviation was calculated (window length: 50 s). Each data value of StO_2_, SpO_2_ and PR was then deleted from the respective time series (set to “NaN”) as soon as a threshold value (*T*) exceeded the moving standard deviation. The value of *T* was determined empirically for each data set to ensure that all artefacts were removed from the data.

During data processing, it was found that the changes in the physiological time-series were quite different for each experiment or neonate. Therefore, each data set from each subject was first examined to determine whether the first CMT intervention (Interval 3 (10–20 min)) caused a statistically significant (*p* < 0.05, Wilcoxon rank sum test) change in StO_2_ in the auditory cortex. In addition, it was determined whether the change was a decrease or an increase in StO_2_.

Block-averages of the data were calculated by normalizing the data to the median of the second interval (touching, 5–10 min), the interval preceding the first CMT intervention (interval 3), and calculating the median as well as the 25th and 75th percentiles of the distribution.

Generalized additive models (GAM), an extension of generalized linear models, were used to investigate the relationship between changes in StO_2_ (AC, PFC) and systemic physiology (SpO_2_ and PR). The following GAM were used: *g*(ΔStO_2_ (AC)) = *β*_0_ + *f*_ts_(ΔSpO_2_, ΔPR) + *ε* and *g*(ΔStO_2_ (PFC)) = *β*_0_ + *f*_ts_(ΔSpO_2_, ΔPR) + *ε*, where the Δ symbol refers to CMT-induced changes in the respective biosignal, *β*_0_ the intercept, *f*_ts_ a tensor product smooth function, *g* a smooth monotonic link function and *ε* the residual. The models were fitted by penalized likelihood maximization.

Artifact removal, signal filtering and block averaging were performed in Matlab (version 2019a, The Mathworks, Natick, Massachusetts). Statistical analysis was performed in Matlab, R (version 4.1.2, R Foundation for Statistical Computing, Vienna, Austria) and JASP (version 0.10.0.0). Figures were created using Matlab, R, Adobe Illustrator (version CS6, Adobe systems software, Ireland) and Gimp (version 2.10.4).

The guidelines of the Society for Functional Near-Infrared Spectroscopy^59^ were followed for the design of the study and the description of the study results.

## Results

### The CMT-induced changes in cerebrovascular oxygenation and hemodynamics showed two types of responses

**Fig. 2:**
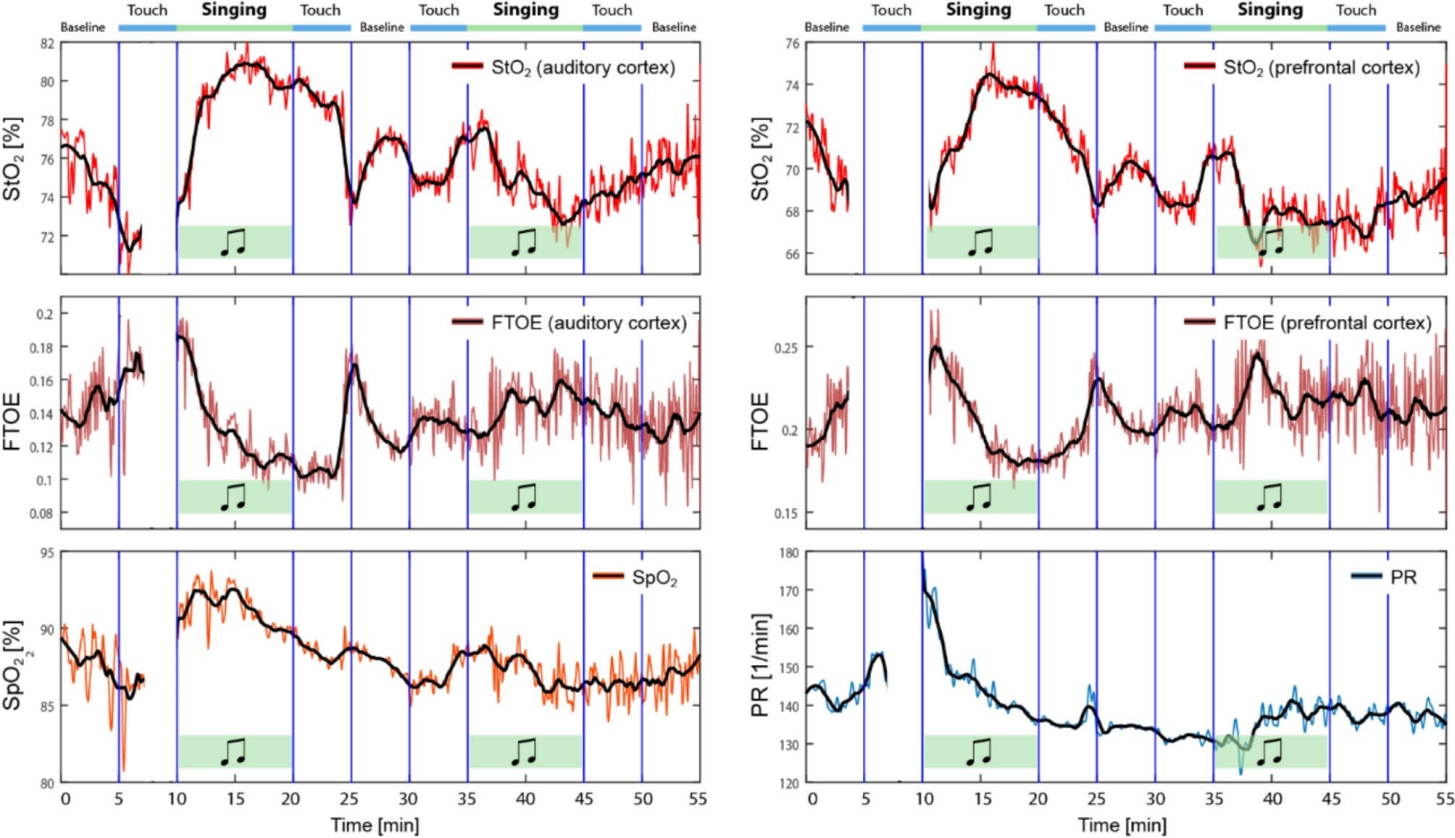
Example of CMT-induced changes in cerebral tissue oxygenation and metabolism as well as systemic physiological signals in one single neonate with a particularly marked change in all physiological signals. StO_2_: cerebrovascular oxygenation, FTOE: dependent fractional tissue oxygen extraction, SpO_2_: peripheral arterial oxygenation, PR: pulse rate. The optodes of the NIRS-based tissue oximeter were placed over the right prefrontal cortex and left auditory cortex.

Some newborns showed a marked change in physiological signals during CMT (see Fig. 2 for an example of the data from one neonate). The response pattern showed a marked increase in StO_2_ and a decrease in FTOE in both cortices during the first phase of singing, in parallel with an increase in SpO_2_ and PR. During second phase of singing, this pattern was much smaller and also more variable.

**Fig. 3:**
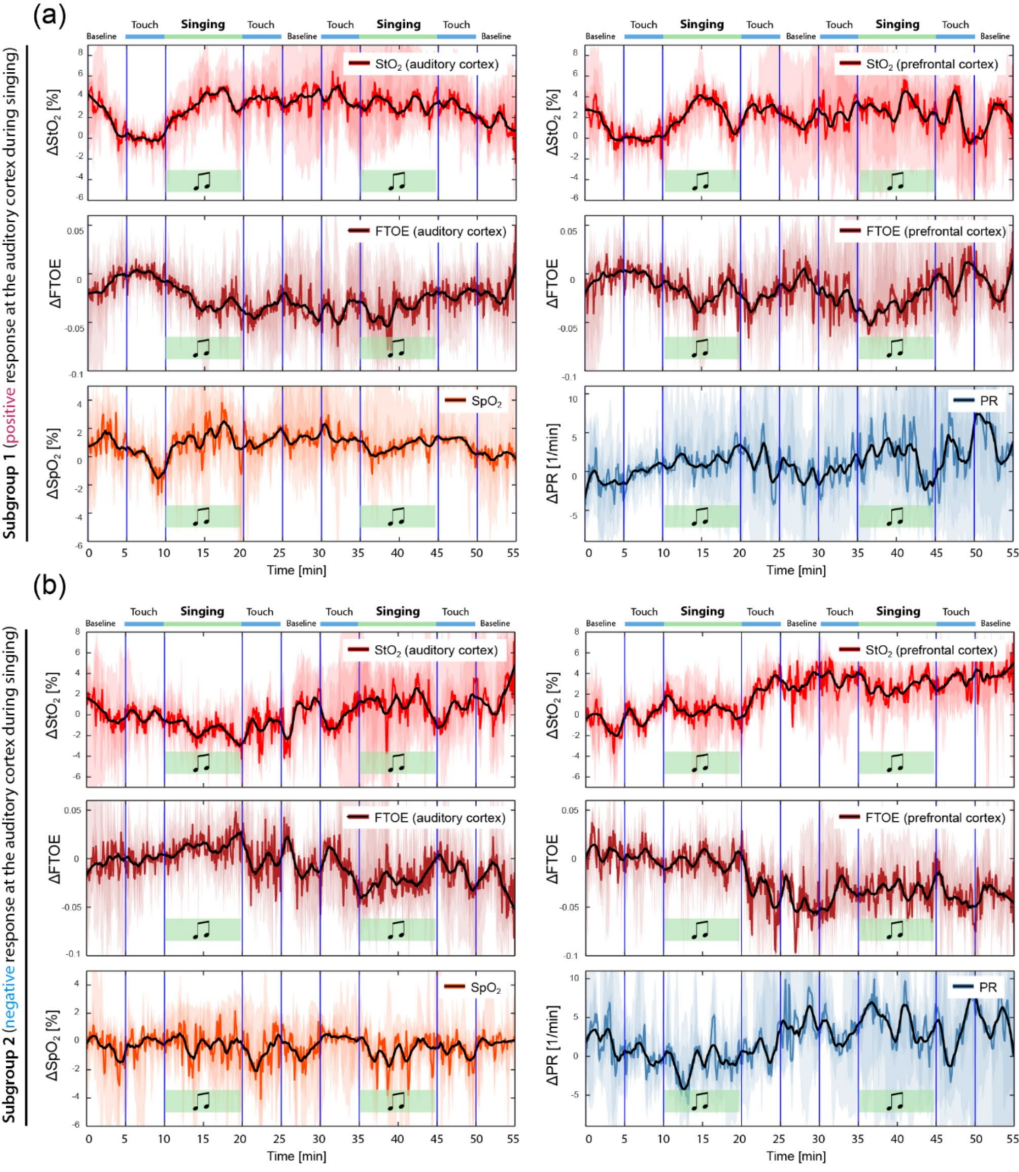
Group-averages of CMT-induced changes in cerebral tissue oxygenation and metabolism as well as systemic physiological signals, depending on the two subgroups. StO_2_: cerebrovascular oxygenation, FTOE: dependent fractional tissue oxygen extraction, SpO_2_: peripheral arterial oxygenation, PR: pulse rate. The optodes of the NIRS-based tissue oximeter were placed over the right prefrontal cortex and left auditory cortex.

After analysing the individual CMT-induced changes of the biosignals for each neonate, we noticed a pattern: each neonate showed either an increase or decrease in StO_2_ in the auditory cortex during the first period of singing. In 94% (16 out of 17) of the neonates, this increase or decrease was statistically significant (*p* < 0.05). Based on this observation, we divided the data sets into two groups: subgroup 1 (positive response) and subgroup 2 (negative response). Figure 3 shows the group-average across all neonates for each subgroup. The following can be observed: In subgroup 1, there is an increase in StO_2_, SpO_2_ and PR, and a decrease in FTOE during the first phase of singing. In subgroup 2, an opposite pattern is observed for all these parameters.

**Fig. 4:**
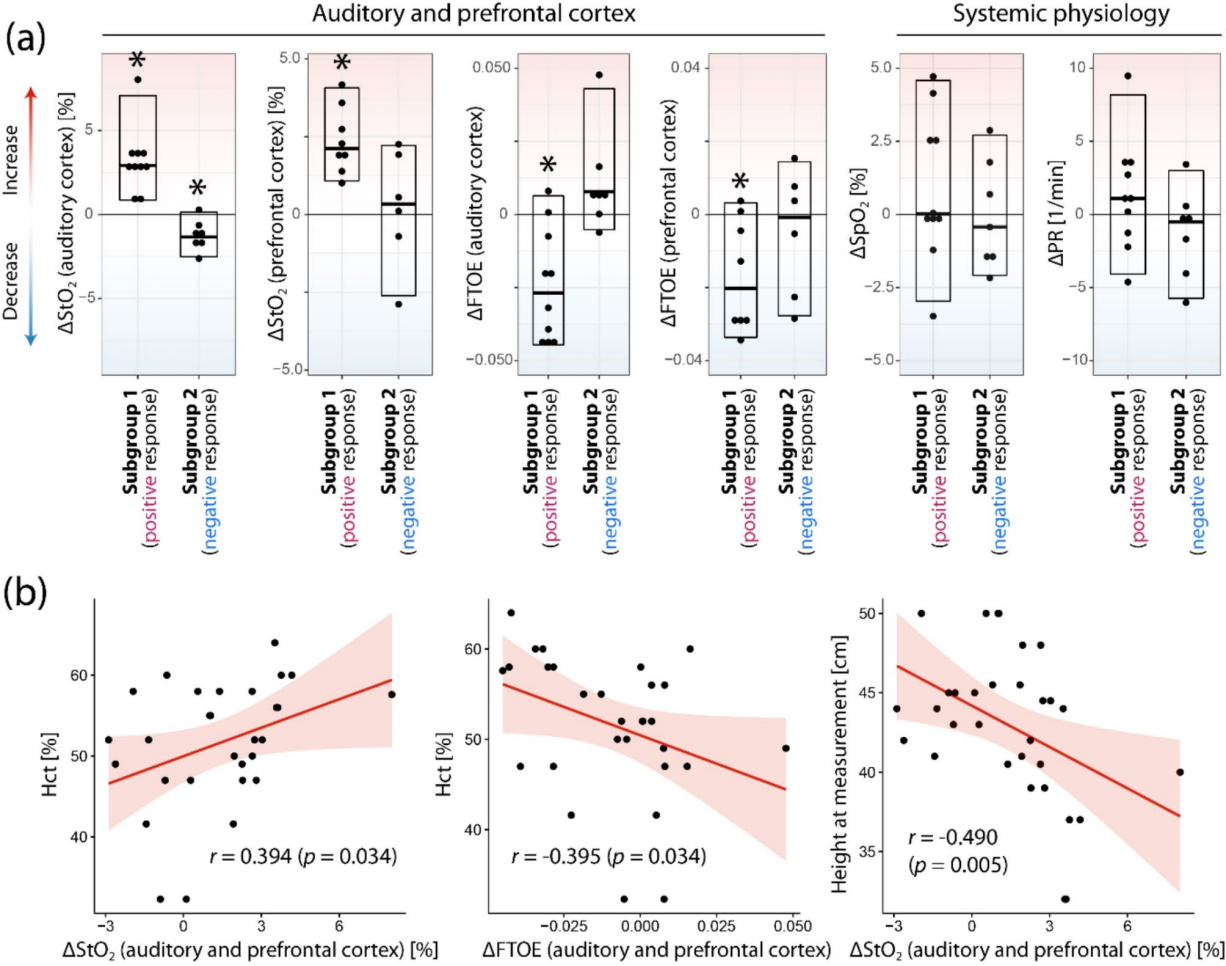
(**a**) CMT-induced changes in cerebral oxygenation and hemodynamics as well as systemic physiology. Asterisks above the boxplots indicate a statistically significant (*p* < 0.05, Wilcoxon signed-rank test) difference from baseline (first touch). (**b**) Statistically significant correlations between haematocrit (Hct) and ΔStO_2_ and ΔFTOE as well as ΔStO_2_ and height at measurement.

A statistically significant change (*p* < 0.01, Wilcoxon signed-rank test) was observed for subgroup 1 in StO_2_ in auditory cortex (*p* = 0.002, increase of 3.2 ± 2.0 SD % StO_2_, effect size (rank-biserial correlation): *r*_rb_ = 1.00 (95% CI: [1.0, 1.0])) and prefrontal cortex (*p* = 0.008, increase of 2.4 ± 1.1% StO_2_, *r*_rb_ = 1.000 (95% CI: [1.000, 1.000])), as well as in FTOE at the auditory cortex (*p* = 0.014, decrease of 0.024 ± 0.019 units, *r*_rb_ = -0.855 (90% CI: [-0.962, -0.518])) and prefrontal cortex (*p* = 0.039, decrease in 0.017 ± 0.015 units, *r*_rb_ = -0.833 (95% CI: [-0.962, -0.398]). In subgroup 2, only StO_2_ at the auditory cortex showed a statistically significant change (*p* = 0.03, decrease in 1.2 ± 0.9% StO_2_, *r*_rb_ = -0.929 (95% CI: [-0.986, -0.675])). Changes in SpO_2_ and PR were not statistically significant in either subgroup (*p* > 0.05). See Fig. 4a for a visualization of the results. Changes in StO_2_ between subgroup 1 and 2 were statistically significantly different (Welch’s *t*-test) for the auditory cortex and prefrontal cortex (*p* < 0.001 and *p* = 0.037, respectively) as well as for FTOE of the auditory cortex (*p* = 0.002).

### Female neonates showed different CMT-induced changes in cerebrovascular oxygenation and hemodynamics compared to male neonates

We investigated which demographic factors and baseline conditions (in terms of brain physiology and systemic physiology) might distinguish the two subgroups. The analysis showed that both subgroups differed with respect to the factor “sex” (*p* < 0.05) (validated with a *Χ*^2^ test of the contingency table; *Χ*^2^ = 4.496, *df* = 1, *p* = 0.034, log odds ratio: -2.303 (95% CI: [-4.559, -0.046]), Kandall’s Tau-b = -0.514, *Z* = -2.057, *p* = 0.040). Subgroup 1 (positive response) included more female neonates (female *n* = 8, male *n* = 2), whereas subgroup 2 (negative response) included more male neonates (male *n* = 5, female *n* = 2). Other factors, such as the APGAR score (which describes the condition of the newborn immediately after birth^60^), age or the behavioural state, did not differ between the subgroups (Table 1).

**Table 1:** Results of the statistical analysis (Mann-Whiteney U test) of possible differences in demographic parameters and baseline cerebral physiology and systemic physiology. *W*: test statistic, *p*: *p*-value, *r*_rb_: rank-biserial correlation (a measure of effect size), AC: auditory cortex, PFC: prefrontal cortex, hct: haematocrit, Apgar: Apgar score reflecting the neonate’s condition after birth. The terminology ⟨.⟩ Indicates that this parameter represents the average.

Parameter	W	*p*	*r*_rb_ (95% CI)
⟨StO_2_⟩ (AC)	39.00	0.740	0.114 (−0.429, 0.597)
⟨StO_2_⟩ (PFC)	20.000	0.662	−0.167 (−0.664, 0.434)
⟨FTOE⟩ (AC)	37.000	0.887	0.057 (−0.475, 0.559)
⟨FTOE⟩ (PFC)	26.000	0.852	0.083 (−0.500, 0.614)
⟨SpO_2_⟩	45.000	0.364	0.286 (−0.273, 0.700)
⟨PR⟩	32.000	0.813	−0.086 (−0.578, 0.453)
Apgar 1 min	45.500	0.255	0.300 (−0.258, 0.708)
Apgar 5 min	40.000	0.606	0.143 (−0.405, 0.615)
Mode of delivery	35.500	1.000	0.014 (−0.508, 0.529)
Hct	18.000	0.168	−0.429 (−0.780, 0.129)
Sex	53.500	0.046 *	0.514 (−0.005, 0.815)
Gestational age	25.000	0.353	−0.286 (−0.700, 0.273)
Postnatal age	40.500	0.623	0.157 (−0.393, 0.624)
Weight (at birth)	31.000	0.740	−0.114 (−0.597, 0.429)
Weight (at measurement)	29.500	0.625	−0.157 (−0.624, 0.393)
Length (at birth)	35.000	1.000	0.000 (−0.518, 0.518)
Length (at measurement)	44.000	0.406	0.257 (−0.301, 0.684)
Head circumference (at birth)	28.000	0.522	−0.200 (−0.651, 0.355)
Head circumference (at measurement)	27.000	0.461	−0.229 (−0.668, 0.328)
Behavioural stage (mean)	38.000	0.807	0.086 (−453, 0.578)
Behavioural stage (STD)	35.000	1.000	0.000 (−0.518, 0.518)

### Individual blood haematocrit concentration correlated with the magnitude and sign of CMT-induced changes in cerebrovascular oxygenation and hemodynamics

To gain further insight into the reasons for the two subgroups, i.e. the between-subject variability of the physiological responses elicited by CMT (i.e. the first singing period of CMT), a correlation analysis was performed to investigate possible relationships between the magnitude (and sign) of the physiological responses and demographical and physiological baseline variables. For the analysis, the values from the auditory and prefrontal cortex were considered together. Individual blood haematocrit (Hct) was found to correlate with the individual magnitude of changes in StO_2_ (Spearman correlation *r* = 0.394, *p* = 0.034) and FTOE (*r* = -0.395, *p* = 0.034). In addition, the length of the neonate at measurement also correlated with the individual magnitude of changes in StO_2_ (*r* = -0.490, *p* = 0.005). See Fig. 4b for the scatter plots of the correlated parameters.

### Subgroup-dependent coupling between cerebrovascular oxygenation and systemic physiology

**Fig. 5:**
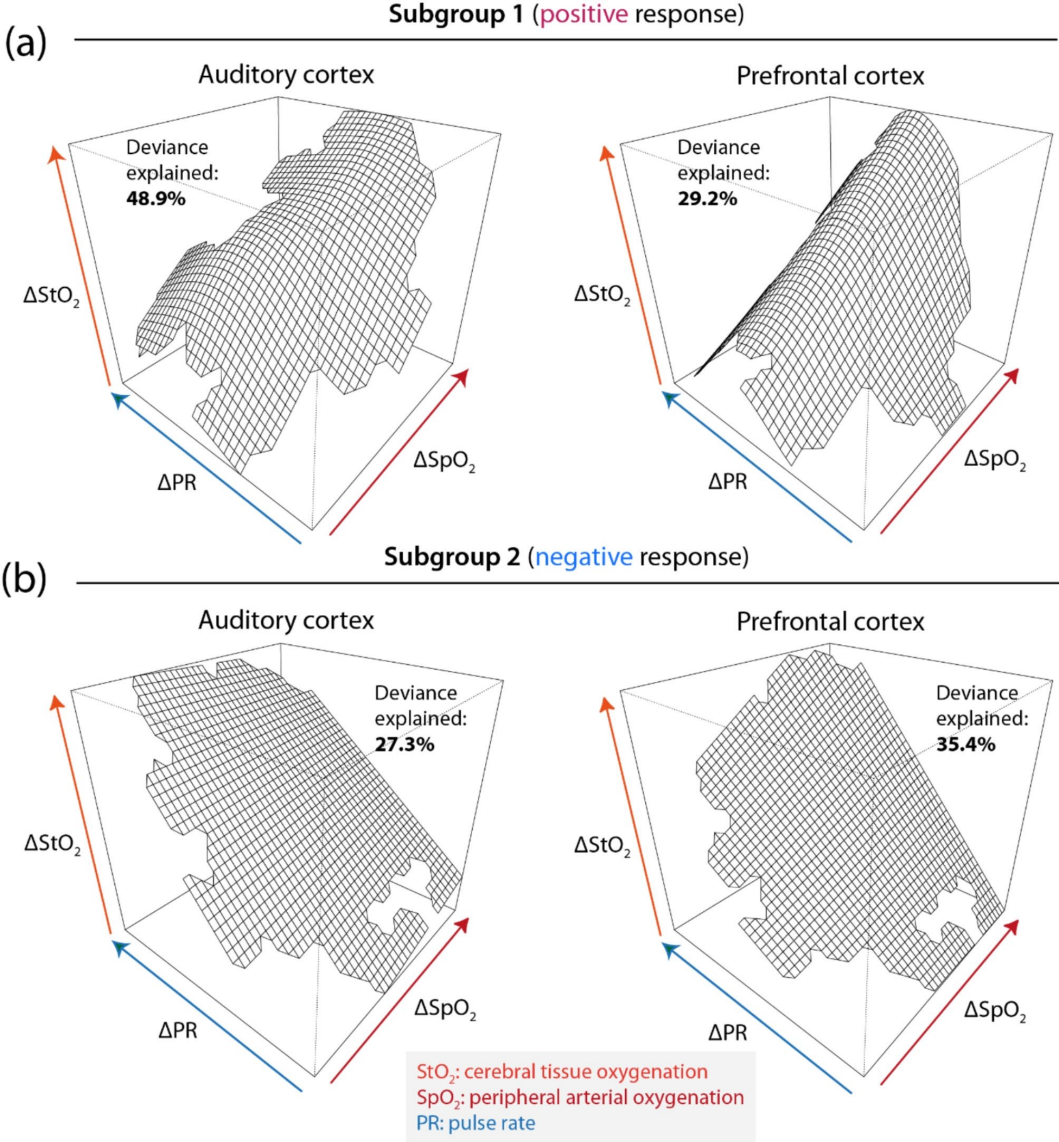
Results of four GAM models linking cerebrovascular oxygenation at the auditory and prefrontal cortex to systemic physiological activity based on the models *g*(ΔStO_2_ (AC)) = *β*_0_ + *f*_ts_(ΔSpO_2_, ΔPR) + *ε* and *g*(ΔStO_2_ (PFC)) = *β*_0_ + *f*_ts_(ΔSpO_2_, ΔPR) + *ε*. The models were evaluated for each of the two subgroups separately (**a**, **b**). It can be clearly seen that the relationships between ΔStO_2_ and ΔSpO_2_ as well as ΔPR are different for each subgroup.

The GAM-based investigation of how the temporal evolution of StO_2_ in auditory cortex and prefrontal cortex relates to systemic activity revealed that the correlations were different for the two subgroups: (i) subgroup 1 (positive response) was characterized by a monotonic positive relationship between ΔStO_2_ (auditory and prefrontal cortex) and ΔSpO_2_, and a non-linear (quadratic) relationship between ΔStO_2_ (auditory and prefrontal cortex) and ΔPR (see Fig. 5a); (ii) subgroup 2 (negative response) showed a monotonic positive relationship between ΔStO_2_ (auditory and prefrontal cortex) and ΔPR and almost no correlation with ΔSpO_2_ (see Fig. 5b). The model relating ΔStO_2_ in the auditory cortex to ΔPR and ΔSpO_2_ had the highest explanatory power with an explained deviance of 48.9%. The modelled smooth tensor surfaces of the terms ΔPR and ΔSpO_2_ of all four models were statistically significant (*p* < 0.01) factors in explaining the distribution of the ΔStO_2_ data.

## Discussion

The aim of our study was to further explore the physiological effects of CMT in preterm infants. To this end, we investigated whether CMT alters cerebrovascular oxygenation and perfusion in the left auditory and frontal cortex and how this relates to baseline and evoked changes in systemic physiological parameters.

### CMT-induced changes in cerebrovascular oxygenation, hemodynamics and systemic physiology

We found that CMT leads to characteristic physiological responses in the preterm infants, with two types of responses found with respect to the change in StO_2_ in the auditory cortex during the first CMT session: neonates with an increase in StO_2_, SpO_2_ and PR (subgroup 1), and neonates with the opposite pattern (subgroup 2). The variability of physiological responses between neonates therefore follows two different types of responses. Interestingly, the same finding was recently made in an fNIRS study by van Dokkum et al.^39^, who also investigated music therapy in preterm neonates (*n* = 20, gestational age: 27.4 (26.3–29.6) weeks, postnatal age: 24.5 (16.5–29.8) days, 44 music therapy sessions). The authors measured StO_2_ and FTOE at the frontoparietal side of the head (either left or right) as well as SpO_2_ and PR during music therapy with improvised music (with instruments and voice; duration of the interval with music: 15 min). Two distinct responses were observed: either an increase in StO_2_ and a decrease in FTOE, or a decrease in StO_2_ and an increase in FTOE—the same result as we found in our study. In the fNIRS study by Meder et al.^40^ investigating the effect of maternal singing combined with skin-to-skin care (duration of the interval with music: 20 min) on StO_2_ measured over the temporal region in preterm infants (*n* = 31, gestational age: 30^26,32^ weeks; postnatal age: not reported), a group difference was not investigated but it was found that on group average there was an increase in StO_2_ during the music intervention (as we observed in subgroup 1 in our study). There was no significant change in FTOE. It would be an interesting project to re-examine the data from Meder et al. to see if there are indeed two subgroups of response.

Remarkably, we also found different hemodynamic response patterns in the visual cortex of preterm infants in another study we conducted in which we examined responses to relative visual stimuli (duration: 35.8 ± 3.4 s)^27^. We found that the responses could be classified into three groups according to the changes in oxyhemoglobin (O_2_Hb]) concentration (subgroup 1: increase, subgroup 2: decrease, or subgroup 3: inconclusive).

What is the physiological reason why neonates can show different stimulus-evoked hemodynamic response patterns? In particular, an answer must be found as to why the preterm-born neonates in our current study and also in the study by van Dokkum et al.^39^ showed two different response patterns to music therapy. In the present study, we found that the two subgroups differed in terms of the composition of the two sexes (subgroup 1 [positive responses in StO_2_]: more females), and that StO_2_ and FTOE correlated with Hct, and ΔStO_2_ with the length at measurement. In the fNIRS study by van Dokkum et al.^39^, the two subgroups did not differ in all the demographic and medical variables characterizing the groups (e.g. age, sex). Unfortunately, the Hct and length at measurement were not included in the analysis by this group. Regarding our previous fNIRS study with neonates and short visual stimuli and the identification of three subgroups, we observed that the three subgroups differed in weight at measurement and Hct. Subgroup 1 (positive response) had a lower Hct and a higher weight than subgroup 3 (no response). However, a statistically significant correlation between ΔO_2_Hb and Hct was not found. A fNIRS study with newborns by Zimmermann et al.^61^ demonstrated a correlation between the strength/sign of hemodynamic responses (Δ[O_2_Hb]) to brief auditory stimulation and the total hemoglobin (tHb) concentration (which is linearly correlated with Hct): positive hemodynamic responses were more likely to be seen in neonates with higher tHb concentrations. This is consistent with the results of our study (considering that Δ[O_2_Hb] and ΔStO_2_ are positively correlated). Zimmermann et al. concluded that the transition from fetal (HbF) to adult hemoglobin (HbA) as well as the age-dependent low Hct may affect neurovascular coupling^61^. As shown by our group, hemoglobin concentration and Hct increase non-linearly with gestational age (22–42 weeks) and decrease non-linearly with postnatal age (0–28 days)^62^. The concentration of HbF decreases and that of HbA increases with gestational age^63^. Since an increase in HbF is associated with changes in the relationship between the partial oxygen pressure (PaO_2_) and arterial oxygenation as measured by pulse oximetry (SpO_2_) (i.e. a leftward shift in the oxygen-hemoglobin dissociation curve)^64^, the individual HbF/HbA ratio in a neonate is also likely to influence the characteristics of the stimulus-evoked changes in StO_2_ (and FTOE). We expect that these aspects play a role in explaining the subgroups in terms of hemodynamic responses. In addition, the maturity of hemodynamic regulatory processes other than neurovascular coupling (i.e. cerebral autoregulation and vasoreactivity) may also play a role. For example, dynamic autoregulation, is underdeveloped in preterm neonates^65^.

Changes in StO_2_ and FTOE should be primarily due to changes in cerebral blood flow (CBF), cerebral blood volume (CBV) and cerebral metabolic rate of oxygen (CMRO_2_), as a direct consequence of neurovascular coupling^66–68^, with components of the signals also being caused by changes in the extra-cerebrovascular oxygenation and hemodynamics, as well as systemic physiology, i.e. changes in the state of the autonomic nervous system (which can, for example, cause vasoconstriction or vasodilation) or changes in blood pressure^51,53,69^. Our measured changes in StO_2_ and FTOE are therefore a mixture of two causes (the ratio of which is not directly known): neurovascular coupling (and hence brain activity) and processes not induced by neurovascular coupling. The fact that changes in systemic physiology have indeed occurred due to CMT is clearly evident from the observed changes in SpO_2_ and PR. The dynamic coupling of ΔStO_2_, ΔSpO_2_ and ΔPR (especially in subgroup 1) that we also observed (Sect. 2.4) further supports this conclusion. The fact that we also observed a fairly synchronous change in StO_2_ and FTOE during CMT in the auditory and prefrontal cortex (especially in subgroup 1) is a further confirmation of this conclusion, as a change caused solely by neurovascular coupling would have been expected primarily in the auditory cortex. As shown in the important review by Kozberg et al.^68^, changes in oxygenation hemodynamics in the developing brain “are particularly susceptible to stimulus-evoked systemic blood pressure increases, leading to cortical hyperemia that resembles adult positive BOLD [blood oxygen level-dependent] responses”, which then represents a confound that “may account for much of the variability in prior studies of neonatal cortical hemodynamics.” These findings by Kozberg and Hillman are probably also relevant in explaining why the first interval of singing in our study showed a much more significant change in physiological parameters. The significant changes in SpO_2_ and PR indicate that changes in systemic physiology have occurred that are probably not directly attributable to neurovascular coupling, but rather to changes in the cardiovascular and/or autonomic nervous systems. It should also be noted that long stimulation times (as in our case of 10 min) are not usually used in fNIRS studies^37,70,71^ and arguably have an additional potential to stimulate systemic physiology—a finding that members of our research group (FS, HZ) have also arrived at through SPA-fNIRS studies in adults using long-term (10 min) visual (colored light) stimulation (with and without cognitive task in parallel)^54,72–76^. Interestingly, in these studies we were also able to show that the individual responses in the SPA-fNIRS signals can be assigned to specific subgroups based on their characteristics^72,74,76^—similar to the result obtained by the present study.

Another possibility for the two groups of different stimulus-evoked hemodynamic response patterns observed in our study could be individual differences in music-induced brain activity in the infants. Such individual differences could for example reflect different experiences or patterns of brain maturation during the prenatal period or during life after birth and before measurement. For example, exposure of preterm newborns to womb-like maternal sounds has been shown to influence auditory cortex maturation (compared to environmental noise)^77^. Individual differences in prenatal stress exposure, which affects the developmental programming of the stress response involving the hypothalamic-pituitary-adrenal axis^78^, may also have shaped the magnitude and type of brain activity response in CMT as observed in our study. Whether such an effect plays a role in explaining our results cannot be assessed from the available data, but could be a topic for further research.

To provide an even better physiological explanation of the dynamics and coupling of the physiological parameters measured in our study, it may be useful to use computational physiological modelling of brain physiology^79–82^, as we have already shown with fNIRS datasets from adults^69^ and preterm neonates^83^. We will address this issue separately in a follow-up study.

The fact that our two subgroups differed in sex ratio (more females in subgroup 1) is an interesting observation, but could still be purely coincidental, as our total number of subjects was small and the effect size relatively small (associated with a *p*-value just below the [controversial^84^ *α* = 0.05 threshold). On the other hand, in our fNIRS study with adults, we also found differences in hemodynamic response with respect to sex^85^. The effect of sex on the hemodynamic responses was also observed in an fNIRS study by Kameyama et al.^86^. Furthermore, it has been shown that female preterm neonates generally have a lower absolute CBF compared to males^87^. In addition, there are sex differences in hormonal function and stress responses are evident in neonates^88^, which may also be relevant to cerebral neurovascular regulatory processes.

The attribution of length requires further research. We assume that length and/or weight are the most likely parameters to correlate with the neonate’s state with respect to its (in utero, ex utero) development and physiological constitution. Future research is needed to investigate in detail the physiological reason for this correlation we have found.

### Strengths and limitations

Our study has several strengths, including (i) the use of a reliable and accurate NIRS-based cerebral oximetry measurement device^49^, (ii) the use of a structured, well-implemented and studied live CMT approach for preterm infants^47^, (iii) the use of the SPA-fNIRS methodology, which allows for the measurement of SpO_2_ and PR along cerebrovascular oxygenation and perfusion, (iv) a data analysis involving elaborate signal processing, statistical analysis (with a special focus on subgroup effects) and statistical modelling.

Concerning possible limitations of our study, we believe that the main one is the number of clinically stable preterm-born neonates measured (*n* = 20); a larger number would have added even more statistical power to our results; however, it must also considered that fNIRS measurements in neonates are associated with a high level of effort and that our measurements showed clear effects despite the relatively small number of subjects, also with regard to the division into subgroups.

## Summary, conclusion and outlook

### Summary of main results

The aim of our study was to further investigate the physiological effects of CMT in preterm infants. The results of the study can be summarised as follows: (i) CMT induced characteristic physiological changes in the neonates whereas changes in cerebrovascular oxygenation showed two types of responses (group 1: increase in StO_2_ during the first singing interval, group 2: decrease in StO_2_); (ii) female neonates showed different CMT-induced changes in cerebrovascular oxygenation and hemodynamics compared to male neonates (i.e. group 1 [increase in StO_2_] comprised more female neonates); (iii) the individual blood haematocrit concentration correlated with the magnitude and sign of CMT-induced changes in cerebrovascular oxygenation and hemodynamics (i.e. higher Hct associated with an increased likelihood of a positive response in StO_2_ to CMT); (iv) the coupling between CMT-induced changes in cerebrovascular oxygenation and systemic physiology was different in the two subgroups.

### Implications of our study for future fNIRS studies with creative music therapy or other functional tasks

Our study showed that CMT induces characteristic physiological responses in neonates. Our main finding, that these characteristic responses can be divided into two groups, is relevant for all future fNIRS studies in neonates. As our previous fNIRS study in neonates showed for short-term (20 s) visual stimulus-evoked responses^27^, the present fNIRS study observed the same subgrouping effect with long-term (10 min) auditory and tactile (CMT) stimulus evoked responses.

For future fNIRS studies on CMT effects in neonates, it would be interesting to extend the head coverage of the fNIRS optodes to cover the whole head and to expand the types of measured physiological signals, for example including the measurement of changes in the concentration of oxidized cytochrome-c-oxidase (as a marker of cerebral metabolism and neuronal activity)^89,90^ and/or CBF using diffuse correlation spectroscopy^91,92^. Measuring the changing state of the autonomic nervous system via electrodermal activity (EDA)^93,94^ would also be of interest.

In future studies, long-term intervention with CMT may show that music processing is beneficial for the development of high-order cognitive, socio-emotional and other functions. Furthermore, CMT is a family-integrating approach and may additionally reduce parental depressive symptoms and anxiety levels and improve physical parent-infant-bonding^95–97^. Therefore, real-time investigations of stress levels (e.g. via EDA measurements), cerebrovascular oxygenation and systemic arterial oxygenation, and possible synchronization processes during CMT in the preterm infants and their parents seem to be of particular interest for further research.

## Data Availability

All data supporting the findings of this study are available within the paper. The datasets used to generate the figures are available from the corresponding author on reasonable request.
